# What Is Cincho-Quinine?

**Published:** 1877-08

**Authors:** Walter A. Taylor

**Affiliations:** Atlanta, Ga.


### WHAT IS CINCHO-QUININE?

By WALTER A. TAYLOR, Ph. G., Attanta, Ga.

The Atlanta Medical and Surgical Journal, for June, contains a communication from Dr. King, headed "Cinchona Alkaloids and their salts." In noticing it, I am reminded of the boy who, finding his aged sire bent over supporting himself, struck him a blow that felled him. The old man, recovering himself, demanded an explanation of his conduct, whereupon the boy said: "You sot so *far* for it I couldn't help it." It would be natural to infer that the writer would, "owing to the scarcity and high price of quinine," advocate the use of one of the cheaper cinchona alkaloids; but in this we were mistaken. He has undoubtedly employed the wrong heading, since his much valued substitute is neither an alkaloid nor an alkaloidal salt of the cinchona bark. He even goes so far as to condemn sulphate cinchonidia, the now best known substitute for sulphate quinine, giving an unqualified indorsement to the nostrum called cincho-quinine, an article which was thrown upon the market several years ago, by Jas. R. Nicholas & Co., now Billings, Clapp & Co., of Boston, Mass. (the ingenious and intelligent pharmacists of Dr. King.) The manner in which the profession was drawn to the nostrum is familiar to all. Not by the "smiles and graces of silver-tongued agents," but by diligent and profuse advertising, the manufacturers and their medical friends have represented it to be an alkaloidal representative of cinchona bark. With this, and the fact of its being much cheaper than the sulphate of quinia, they have "offered gold, and furnished gilded dross." The article has gained favor with many physicians, who administer it as a substitute for sulphate of quinine, but among the pharmacists, especially those who were educated to a professional standard, the nostrum was looked upon with suspicion, and soon after its introdaction was subjected to a chemical examination, in 1870, by Wm. T. Wenzell; in 1874, by Albert E. Ebert; in 1875, by Profs. Scheffer and Diehl, and others. Neither Wenzell nor Ebert were successful in determining the presence of quinia; Profs. Scheffer and Diehl found quinia, quinidia, and probably cinchonidia, in very small quantities; the other analysists succeeded in determining the presence of quinia, quinidia and cinchonia. The amount of each was not stated until 1876, when Mr. E. M. Wells, assisted by Prof. Jno. M. Maisch gave the following results (*American Journal of Pharmacy*, June, 1876,) "using for experiments two samples of cincho-quinine, which will be designated as "new" and "old." The new sample was obtained from the manufacturers in December, 1875; the old sample, July 18, 1873, from a firm in New York. The qualitative examination was conducted in the same manner as detailed by Profs. Scheffer and Diehl, (*American Journal of Pharmacy*, April, 1875,) and gave the same results, proving the presence ef ammonium sulphate, cinchonia sulphate, and cinchona alkaloids; also, a small quantity of organic matter, which could not be dissolved in alcohol, but remained behind with the ammonium sulphate, and with it was soluble in water."

The results of these assays, calculated for 100 parts of material, were as follows:

| | New sample. | | Old sample. | |
|---|---|---|---|---|
| Sulphuric acid (SO3), . . . . . . . . | 6.516 | | 5.016 | |
| Ammonium (NH4), . . . . . . . . . | 1.360 | | 1.380 | |
| Water, determined by heat, . . . . . . | 4.098 | | 3.174 | |
| Loss (mainly water of crystallization), . . . | 0.359 | | 0.040 | |
| Total non-alkaloid matter, . . . . . . | —— | 12.333 | —— | 9.610 |
| Quinia, . . . . . . . . . . . | 0.466 | | 0.240 | |
| Quinidia, . . . . . . . . . . | 1.250 | | 1.820 | |
| Cinchonidia, . . . . . . . . . | 0.717 | | 0.920 | |
| Resin and loss, . . . . . . . . . | 0.583 | | | |
| Cinchonia, . . . . . . . . . . | 1.384 | | 2.440 | |
| | —— | | —— | |
| Total am't dissolved by first treatment with ether, | | 4.400 | | 5.420 |
| Cinchonia left undiss'lved on first treatm't with ether, | | 83.267 | | 84.970 |
| | | —— | | —— |
| | | 100.090 | | 100.000 |

"The total amount of cinchonia, a portion of which is combined with sulphuric acid, was, therefore, 84.651 per cent. in the new, and 87.410 per cent. in the old sample; while the quinia was present in one case to the amount only of less than one-half, and in one sample less than one-quarter of one per cent."

In connection with the above, I shall also give the remarks of Prof. Jno. M. Maisch. At the close of Mr. Wells' article, he states that:

"It will be of interest to recapitulate, in connection with the above assay, the quantitative results obtained in previous analyses, more particularly the amount of the three higher priced alkaloids, present in cincho-quinine, which form the correct basis for estimating its commercial as well as medicinal value. Wenzell obtained 2.5 per cent., Scheffer and Diehl (see *Amer. Jour. Phar.*, 1875, p. 205,) from four samples, 3.00, 3.25, 2.15 and 3.10 per cent., and Wells (4.400—1.384=) 3.016 and (5.420—2.440=) 2.980 per cent.; the average of these seven assays gives 2.86 per cent. of higher priced cinchona alkaloids, from which figure the lowest amount on record differs by .71 or 25 per cent., and the highest amount recorded by .39 or nearly 14 per cent., while the proportion of the lowest to the highest figure, 2.15 and 3.25, is as 2:3.

A still greater variation is observable in the percentage of quinia, small as it is; the lowest one found (Wells) being ·24, and the highest (Scheffer and Diehl) ·612 per cent., their proportion is nearly as 2:5. Mr. Wells has estimated the amount of ammonium from which the ammonium sulphate in the two samples proves to be 5·44 and 5·52 per cent., representing respectively 3.40 and 3.45 of the sulphuric acid, which deducted from the assayed amounts of the latter, leave 3.116 and 1.566 per cent. of in combination $S\,O_3$ with alkaloids. Allowing this to be combined with cinchonia, we find the following percentage of cinchonia sulphate (which contains 10.69 per cent. $S\,O_3$) in Wells' old sample 14.69, and in his new sample 29.23 per cent., or within a slight fraction, double the amount. The amount of alkaloidal sulphate, approximated by Scheffer and Diehl, by treatment with cold water, varied in four samples between 11.75 and 22 per cent.; a variation nearly as great as found by Mr. Wells. If the figures, as obtained by the different analysists, an average for the various compounds actually contained in cincho-quinine, and the ammonium sulphate be entirely omitted, a preparation closely resembling the *average* composition of the article in question, may be obtained by intimately mixing

| | | | | |
|---|---|---|---|---|
| Quinia, alkaloid, | 5 grains, | or | Quinia sulphate, | 7·5 grains |
| Quinidia alkaloid, | 15 grains, | | Quinidia sulphate, | 20· grains |
| Cinchonidia, Alkaloid, | 10 grains, | | Cinchonidia sulphate, | 12·5 grains |
| Cinchonia, alkaloid, | 770 grains, | | Cinchonia sulphate, | 180· grains |
| Cinchonia, sulphate, | 200 grains, | | Cinchonia, alkaloid, | 780· grains |
| | 1,000 grains. | | | 1,000 grains |

Those who desire a still closer representation, may incorporate with either of the above mixtures 54 grains of ammonium sulphate, which will not enhance the cost but increase the weight of this, as some medical journalists tell us, *valuable* "nostrum."

I shall now endeavor to correct some statements made in Dr. King's paper, but I shall add here, that it is not my purpose to doubt the individual experience of his specific, but we cannot fail to regard it as a little singular, that in giving the thereupeutical effects and chemistry of the article in question, he should so rely upon the statements made by Messrs. Billings, Clapp & Co., in a circular extolling the praises of cincho-quinine. The following are some of his statements:

"Cincho-quinine is a combination of all the active medical principles of the best calisaya bark, etc., etc., possessing all the *advantages* with none of the *disadvantages* of sulphate of quinia, or any of the single alkaloids of the cinchonas. It is entirely free from such external agents as sugar, liquorice, starch, magnesia, etc. It is *wholly* composed of the bark alkaloids, quinia, cinchonia, quinidia, cinchonidia, and other alkaloidal principles which have not been distinctly isolated, and the precise nature of which is not well understood, etc., etc. In its preparation all the active tonic and febrifuge principles of the bark are secured without the inert bulky lignin, gum tannic acid, etc., etc. It exerts the full therapeutic influence of sulphate of quinia, in the *same dose*, without oppressing the stomach or creating nausea. It seldom produces cerebral distress as quinine does, and I have found it to produce much less constitutional disturbance than the latter agent, etc., etc. One of the main points I desire to impress upon the minds of those who read this article is, that cincho-quinine is not sulphate of cinchonidia not sulphate of quinine, but all the alkalids in combination, and it is this *composition*, this representation of all the medicinal principles found in Peruvian bark, that gives it the value claimed for it over and above all other preparations, or any one of the alkaloids," etc., etc.

(Billings, Clapp & Co., in a circular, make the above statement almost verbatim). In answer to the above, I will state that cincho-quinine (as I think I have shown from the analysis) is not a representation of calisaya bark, but to the contrary, it consists mainly of cinchonia and its sulphate, and in one case was found to consist of less than one half, and in another less than one quarter of one per cent. of quinia. It does contain sulphuric acid and sulphate of ammonium, consequently does contain substances not naturally existing in cinchonia bark. It does contain a portion of alkaloids in acid combination, chief among which is sulphate of cinchonia, which in connection with the alkaloid cinchonia amounts to about 85 to 87 per cent.

Again, it is singular that he should be so down upon some of our leading manufacturing chemists. There are but three in this country that manufacture the cinchona alkaloids, and I am sure that neither one of them concede to themselves the praise of being the discoverer of any of the cinchona alkaloids, and will never do so foolish a thing as to attack that specific quinine. They offer simply a substitute, cinchonidia, an alkaloid first obtained and characterised under the name of quinidine (quinidia) in 1847, by F. L. Winckler, of Darmstadt, and in 1852, it was more closely studied in Liebig's laboratory by Leers, still under the same name, so we see that it was not discovered prior to 1835. Supernumeraries are never called in when the originals are so easy of access. Quinine will be used by those who are able to take it as long as it is manufactured. Physicians who have reputation at stake, and when there is a call for THE ANTI-PERIODIC, will, until reason deserts the votaries of medicine, prescribe it, despite the assertion of an occasional M.D., Ph.G., or manufacturer, who says it is too "*expensive.*"

Bare assertions never yet dimmed the reputation of a fact which had for its foundation practical experience. The experience of over a thousand M.D.'s in these United States, is at variance with Dr. King, in his assertions, that it requires three or four times as much sulphate of cinchonidia, as of sulphate of quinine, to secure the "symptoms of true quininism," and fully as many of the honored fraternity bear testimony that as an antiperiodic, it is *not* perfectly worthless, unless given in three or four times the dose of sulphate of quinia.

